# ^1^H-NMR-based metabolomic profiling and proteomic analysis of soybean (*Glycine max* L.) in response to dicarboxylic acids (photon) application as a stress priming agent

**DOI:** 10.1016/j.heliyon.2024.e37466

**Published:** 2024-09-07

**Authors:** Mhlonipheni Nhlakanipho Msomi, Gerhard Prinsloo, Noluyolo Nogemane

**Affiliations:** Department of Agriculture and Animal Health, Florida Science Campus, University of South Africa, Johannesburg, Gauteng Province, South Africa

**Keywords:** Dicarboxylic acids, Stress priming agent, ^1^H-NMR-Based metabolomics, Gas chromatography coupled with mass spectrometry (GC-MS), Proteomics, Soybean

## Abstract

Soybean (*Glycinemax* L.) serves not only as food for humans, animals, and industrial purposes, but is also a plant that can be used to comprehend molecular mechanisms occurring in stress response to various development techniques. To reveal the effect of applying dicarboxylic acids as stress priming agents on a metabolic level in soybean leaf extracts, the chemical profile of methanolic extracts were collected at different time points (1 h, 2 h, 12 h, 24 h, 1 week, 2 weeks and 3 weeks) after spraying were analyzed using ^1^H-NMR based metabolomics by way of PCA and OPLS-DA. The OPLS-DA revealed several metabolites, including organic acids (fumarate, citrate and malate) and amino acids (asparagine, alanine and GABA), which accumulated in higher amounts, with fumarate accumulating the highest in *Glycinemax* L. leaf extracts compared to untreated leaves. Denaturing 1DE gels were prepared for MS-based protein analysis and the presence of fatty acids (linolenic, oleic and α-linolenic acid) were confirmed by gas chromatography coupled with mass spectrometry (GC-MS), which served as jasmonic acid precursors. The MS-based profiling of proteins on the denaturing 1DE gels revealed several proteins that were differentiated between the treated and untreated leaf extracts. These proteins included ferritins, CaM, ferredoxin-thioredoxin reductase and chalcone-flavanone isomerase 1A. Following the treatment, fumarate was significantly elevated at 12 h to 3 weeks, compared to other compounds. It is, therefore, proposed that elevated quantities of fumarate could be related to the KEAP1-NRF2 metabolic pathway. This study represents the initial investigation of the effect of dicarboxylic acid application as a stress priming agent on *Glycinemax* L. using ^1^H-NMR metabolomic analysis, GC-MS and proteomic analysis.

## 1 Introduction

Several physical factors, including tissue types, developmental stages, environmental stimuli, and stresses, continue to be elucidated to comprehend the underlying mechanism of how plants respond to different environmental stresses. As a result of their sessile lifestyle, plants must activate a variety of defense mechanisms against external factors to survive [1]. Developing new insights into the response of plants to genetic and environmental changes induced by biotic or abiotic stresses is unavoidably required in the pursuit of new trait-enhanced crops [2]. Environmental stress reduces crop growth and productivity around the world, which is why it is a global problem [3] and various studies have been conducted in the past with the aim of stress tolerance enhancement in plants. Soybean is one of the important legume crops cultivated in both temperate and tropical environments.

While the primary objective in agriculture is to reduce growth limiting factors to increase crop yields, environmental stresses such as drought, heat and cold continue to have a significant impact on soybean growth and development. Consequently, the search for new crop enhancements contributes to the development of new insights into plant stress response. Various stress priming agents are available commercially, with the aim of preparing the plant for stress stimuli. These priming agents aim at reducing the effect of stress on plants, therefore limiting negative effects such as reduced yield and susceptibility to disease.

Photon is a commercial product that contains dicarboxylic acids and has been developed to enhance the tolerance of plants to environmental stress by acting as a stress priming agent. It involves spraying plants with Photon which is a composition of dicarboxylic acids for improving crop productivity and thus increasing tolerance to environmental factors, such as excess heat, cold, light, soil salinity and water stress (CMM, 2015). Abiotic stresses and adaptation processes elicit responses at multiple levels of plant organization, including the molecular, cellular, physiological and biochemical levels [4]. Molecular responses to stress in plants include altered gene expression, protein abundance and metabolite accumulation [5]. Metabolites are end-products that are produced downstream of gene and protein activity, and their composition and content directly affect the phenotype.

Metabolomics is a systematic method for qualitative and quantitative analysis of many metabolites to better understand how complex metabolic networks interact and how they change under stress adaptation and tolerance [6]. It is one of the tools that uses a variety of techniques to elucidate plant mechanisms and their response to biotic, abiotic stresses and priming agents. It has been used successfully in numerous crops, including soybean [1]. In a study conducted by Ref. [7] on soybean leaves, gas chromatography (GC)- and liquid chromatography (LC)-coupled with mass spectrometry (MS) revealed distinct amounts of primary and secondary metabolites in response to heat and drought stress.

NMR-based metabolomics is one of the techniques that supports metabolic analysis of living samples. It provides distinct advantages compared to other spectroscopic instruments as it is capable of measuring metabolites, such as proteins (lipoprotein particles) that cannot be detected using LC-MS and GC-MS [8]. Additionally, the NMR technique is known for its high reproducibility in comparison to other LC-MS and GC-MS methods. The necessity of investigating proteins that are present after Photon application was guided by the NMR-based metabolomics analysis. Structural proteomics, on the other hand, provides biochemical and cellular functions for unannotated proteins. Therefore, a combination of NMR-based metabolomics and proteomics assist in understanding metabolites and stress proteins in relation to the response of soybean plants to Photon application. In addition, no scientific research that investigates how dicarboxylic acids such as Photon creates stress tolerance in soybean is currently available. This study therefore aimed to determine the effect of exogenously applied dicarboxylic acids (Photon) on the metabolome of soybean leaves using ^1^H-NMR metabolomics and to discover proteins involved in plant stress resistance collected at different time points. The outcome of this study will help in the comprehension of the mode of action of chemical processes in soybean and reveal stress proteins that can be used to further comprehend the plant's reaction for crop enhancement.

## 2 Materials and methods

### 2.1 Reagents and buffers

Deuterium water (D_2_O), deuterated methanol (CD_3_OD), Trimethylsilylpropionic acid sodium salt (TSP), potassium dihydrogen phosphate (KH_2_PO_4_) and all NMR consumables were purchased from Merck SA (Pty) Ltd (Sandton, Johannesburg SA). The ReadyPrep protein kit was purchased from Bio-Rad (Pty) Ltd (Rosebank, Johannesburg SA). All proteomics reagents and buffers were purchased from Inqaba Biotech (Muckleneuk, Pretoria, SA). Liquid nitrogen (N_2_) was purchased from Afrox Wadeville Gas and Gear (Germiston, SA.)

### 2.2 Plant material and treatment

Soybean seeds (*Glycine* *max* L. certified cultivar: PAN 3A-173) were donated by a commercial seed company (Pannar), Mpumalanga province in South Africa. This study was conducted at the Horticulture Centre of the University of South Africa (Science Campus, Gauteng Province of South Africa) during the 2020/2021 period. The geographic coordinate of the site is 26.1586° S, 27.9033° E. According to Roodepoort climate, this area receives a mean annual rainfall of about 607 mm, with most rainfall occurring between October and March. A commercial potting mix (Premium) from Garden master was used in the pots to grow the cultivars. The potting mix was composed of coco peat, vermiculite, and earthworm casting. A total of 32 pots (30 cm volume) with 16 replicates of treated plants were randomized and the other 16 replications served as a positive control. All the plants were grown in a polypropylene shade net with a 70 % shade factor.

Photon treatment was applied using Photon 50SG based on 50 % mixture of dicarboxylic acids at a concentration of 16 mg per liter of water using a knapsack sprayer (Auto Gear 16L High Pressure Sprayer) at V6-sixth leaf 2 h after sunrise. After spraying, Leaves were harvested at various time intervals (1 h, 2 h, 12 h, 24 h, 1 week, 2 weeks, 3 weeks) and immediately submerged in liquid nitrogen (N_2_) using a cooled mortar and pestle to stop further metabolic activity. The leaves were freeze-dried for seven days, and the dried samples were stored in a −80 °C refrigerator until use.

### 2.3 NMR analysis

#### 2.3.1 Preparation of extracts

A direct extraction method for untargeted ^1^H NMR based metabolomic analysis was adapted [9]. Untargeted metabolites were extracted using methanolic water (MeOH) (1:1). Fifty mg of fine powder was weighed out for each sample in 2 mL Eppendorf tubes (Sigma-aldrich). The weighed samples were dissolved in 750 μL a potassium phosphate buffer (750 μL, pH = 6) deuterium water (D_2_O) containing 0.01 % internal reference standard 3-(Trimethylsilyl) propionic-2,2,3,3-d_4_ acid sodium salt (TSP) and 750 μL at room temperature; the mixture was vortexed for 1 min to homogenize it, then ultrasonicated for 20 min to remove any precipitates. The supernatant was transferred to a 5 mm NMR tube (Norell, Sigma-aldrich) for ^1^H NMR spectroscopic analysis and subjected to ^1^H NMR 600 MHz spectrometer (Varian Inc., California, USA) recording 32 scans.

#### 2.3.2 Data pre-processing

The MestReNova (14.2 Mestrelab Research, Spain) was used for data processing. The ^1^H NMR spectrum was manually corrected for phasing and baseline, normalization, and peak alignment. Binning was used to extract data.

#### 2.3.3 Data reduction of the ^1^H NMR spectra

To reduce the effect of chemical shift variation due to small differences in pH, the ^1^H NMR spectra 10 ppm were automatically data reduced to 232 integral segments or bins with a width of 0.04 ppm. The region d 4.50–5.06 ppm was omitted from the analysis to eliminate the effects of variation in water suppression. Each segment consisted of the integral of the NMR region to which it was associated. Excel was then used, where for each sample the spectral integrals were scaled to the integrated spectrum's total intensity. The samples were then imported into the SIMCA software package (version 15.0.2, Umetrics, Umea, Sweden) for multivariate analysis.

#### 2.3.4 Multivariate analysis

To make the datasets easier to interpret, the bucketed data was analyzed using the Principal Analysis Component (PCA) and Partial Least Squares Analysis-to Discriminant Analysis (OPLS-DA) multivariate statistical methods. Furthermore, as a regression extension to PCA, OPLS-DA was performed.

#### 2.3.5 Annotation of metabolites

Chenomx NMR suite 9.0 (Evaluation Edition) was used for metabolite verification and the Human Metabolome Database (HMDB) was used for annotation of compounds.

### 2.4 GC-MS analysis

#### 2.4.1 Preparation of extract and gas chromatography time-of-flight mass spectrometry (GC TOF) analysis

The soybean leaf samples were extracted according to the method used by Ref. [10]. Dried leaf samples of 25 mg was weighed and extracted in 2 mL (95 %) of methanol (GC-grade) from each dried material in 2 mL Eppendorf tubes. The tubes were then vortexed for 3 min with a DLAB MX-5 vortex mixer (USA), sonicated for 20 min in a BRANSON 2800 ultrasound bath, centrifuged for 15 min with an Eppendorf 5424 microcentrifuge (AG, Germany) and filtered with a 0.2 mL syringe filter. Then, 1 mL of the supernatant was transferred to 2 mL GC-MS vials (RESTEK) for analysis.

A Leco® 7890B GC chromatograph with a Gerstel multisampler and Chroma time-of flight mass spectrometer (USA) software optimized for a Pegasus®4D mass spectrometer were used for the analysis of the crude extracts and each sample was run twice. The extracted compounds were separated into two columns. The main column (Restek®) had a length of 32 m, an internal diameter of 250 μm and a film thickness of 0.025 μm; the secondary column had a length of 0.790 m with an internal diameter of 250 μm. Analysis of the samples was carried out under the following conditions: a splitless injection of 0.2 mL was used; the injection temperature was 33 °C, held for 3 min, then increased at a rate of 10 °C per minute to the target temperature of 180 °C; without a holding period, the temperature increased at a rate of 40 °C per minute until a temperature of 220 °C was reached. Helium (99.99 %) was the carrier gas used, with a flow rate of 1.0 μL per minute (constant flow). The ionizing energy was 70 eV. The scan rate was 50 scans per second and the mass spectral scan range was 60–600 (MHz). The relative constituent content was expressed as normalized percent peak area. Samples of pure hexane, dichloromethane and ethanol were used as controls and were included and run as blanks when each of the respective crude extract samples were run.

### 2.5 Protein extraction

To identify proteins from leaf tissues of *Glycine* *max* L., the working solution from the ReadyPrep Protein Extraction Kit (BIO-RAD, USA) was prepared as per the manufacturer's instructions. Fresh soybean leaf samples were ground to a fine powder using a mortar and pestle in liquid nitrogen before adding sample buffer. In a 2 mL centrifuge tube, 50 mg of fine powder was weighed, and 1 ml of sample buffer was added per 50 mg weighed. Each sample was then placed on ice and the suspension was sonicated with an ultrasonic probe (Lasec) for 1 min to disintegrate the cells. In between treatments, the suspension was briefly cooled on ice. The homogenate was centrifuged at 16,000×*g* for 20 min at 18 °C to pellet cell debris using a Centrifuge 5702 R (Lasec). The supernatant was removed and transferred to clean tubes.

#### 2.5.1 1D SDS-PAGE

In a 50 mL centrifuge tube, a mixture of 12 % separating gel was prepared whereby 3.270 μL distilled water, 4500 μL acrylamide solution, 2.630 μL of 1.5 Tris-HCL, pH 8.8, 0.1 μL of SDS (10 % w/v), 20 μL ammonium persulphate (APS) and 11 μL of TEMED were added and thoroughly mixed. For a 12 % stacking gel, the following components were added to another 50 mL centrifuge tube: 200 μL of distilled water, 480 μL of acrylamide solution, 900 μL of 0.5 M Tris-HCL, pH 6.8, 100 L SDS (10 % w/v), 20 μL of APS and 10 μL TEMED. The Coomassie blue dye solution was prepared in accordance with the method described by Ref. [11]. Subsequently, the gel was treated with a Coomassie blue dye solution and allowed to stain for 24 h and subjected to destaining by using distilled water. The gel image was obtained with the G. Box Syngene genesnap (Vacutec, South Africa) after destaining.

#### 2.5.2 In-gel digestion

Proteins were digested from gel fractions according to Ref. [12]. Each lane was separated into 5 gel fractions based on band densities and molecular weight range. Briefly, the proteins were reduced in gel with 10 mM DTT in 25 mM NH_4_HCO_3_ for 1 h at 60 °C. Samples were cooled to room temperature, then 100 % acetonitrile was added and incubated for 10 min. The supernatant was discarded and 55 mM iodoacetamide in 25 mM was added to the gel pieces. The reaction proceeded in the dark for 20 min at room temperature. The supernatant was discarded, and gels were dehydrated with 25 mM NH_4_HCO_3_ in 50 % acetonitrile, vortexed and the supernatant was removed. The gel pieces were dried to completeness and freshly prepared trypsin was added; protein digestion was allowed to proceed overnight at 37 °C. The digestion was quenched by adding final 0.1 % formic acid and the samples were dried under vacuum. Dried samples were re-suspended in 2 % acetonitrile and 0.2 % formic acid for MS analysis.

#### 2.5.3 LC-MS analysis

Tryptic peptides from each gel fraction were analyzed using a Dionex Ultimate 3000 RSLC system coupled to an AB Sciex 5600 TripleTOF mass spectrometer. Injected peptides were inline de-salted using an Acclaim PepMap C18 trap column (75 μm × 2 cm; 2 min at 5 μl min^−1^ using 2 % ACN/0.2 % FA). Trapped peptides were gradient eluted and separated on a Waters nanoEase CSH C18 column (75 μm × 25 cm, 1.7 μm particle size) at a flowrate of 0.3 μL min^−1^ with a gradient of 6–40 % B over 15 min (A: 0.1 % FA; B: 80 % ACN/0.1 % FA). The 5600 TripleTOF mass spectrometer was operated in positive ion mode. Data-dependent acquisition was used; precursor (MS) scans were acquired from *m/z* 400 to 1500 (2^+^-5^+^ charge states) using an accumulation time of 100 milli seconds followed by 40 fragment ion (MS/MS) scans, acquired from *m/z* 100–1800 with 20 milli seconds accumulation time each.

#### 2.5.4 Data analysis

Raw data files were searched with Protein Pilot V5.0 software (SCIEX), using a database containing sequences from *Glycine* max downloaded from UniProt (Swiss-Prot on September 7, 2022) and common contaminants. Trypsin was set as the digestion enzyme, cysteine alkylation (iodoacetamide) was allowed as a fixed modification and biological modifications were allowed in the search parameters. A 1 % false discovery rate filter was applied at the protein level for refinement of identifications.

## 3 Results

### 3.1 ^1^H NMR metabolomic analysis

#### 3.1.1 Composition of metabolites of soybean leaves harvested at different time points

**Fig. 1:**
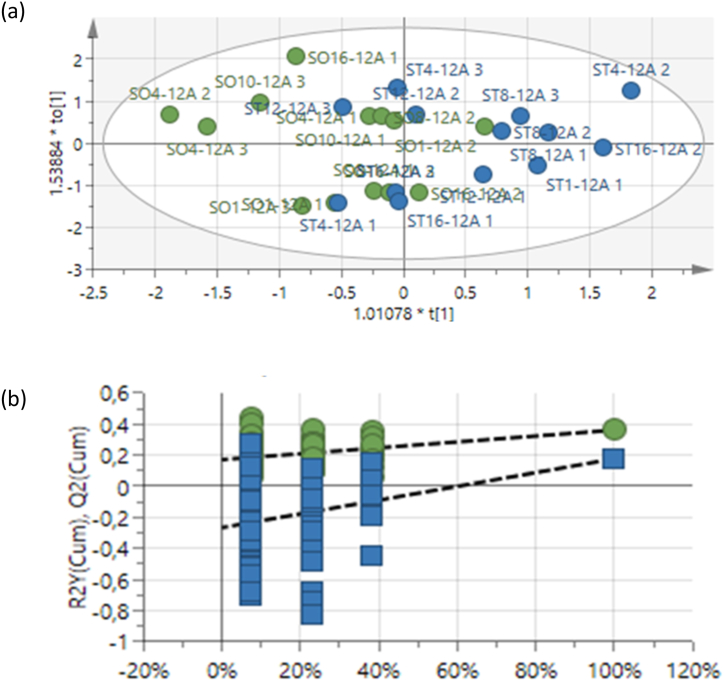
(a) OPLS-DA scores plot showing the predictive (x-axis) and orthogonal (y-axis) components of *Glycinemax* L. leaf extracts collected 12 h following spraying of Photon. Blue dots = treated, green dots = untreated. (b) The 100-permutation test used to validate the model (R^2^X = 0.783, R^2^Y = 0.464, Q^2^ = 0.402).

The ^1^H NMR spectroscopy data of plant metabolic profiles was analyzed to distinguish between treated and untreated *Glycine* *max* L. leaf extracts using the SIMCA software (Umetrics, Umeå, Sweden). The supervised OPLS-DA statistical model was used to compare the leaf extracts of *Glycine* *max* L. in response to the application of Photon. The treatment seemed to be not effective after 2 h of spraying as there was no separation between treated and non-treated samples (not included in the paper). After 12 h of treatment, treated leaf extract samples grouped to the right of the ellipse using OPLS-DA, showing that treated samples were more similar than untreated samples (Fig. 1a). The 100-permutation test was generated to validate the model (Fig. 1b)

**Fig. 2:**
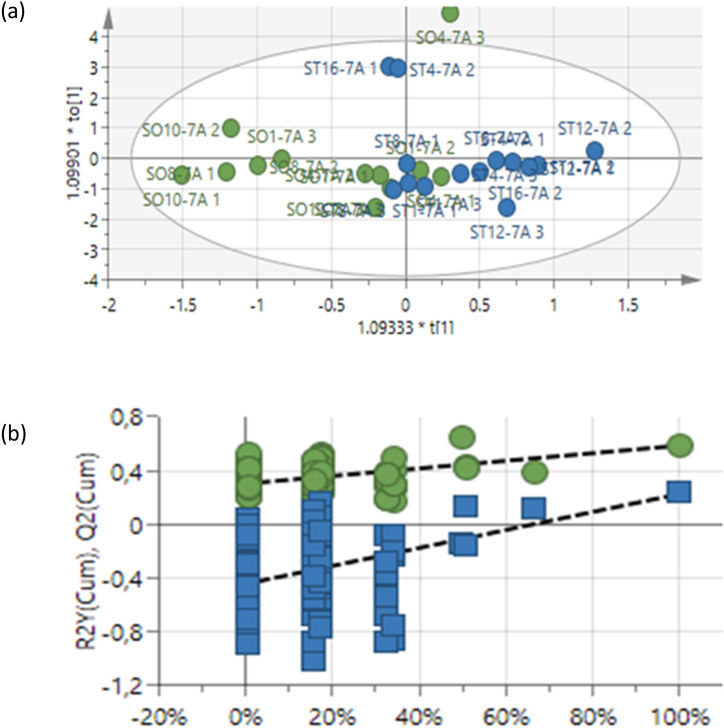
(a) OPLS-DA scores plot showing separation of non-treated (green) and treated (blue) leaf samples collected 1 week after spraying of Photon. (b) The permutation test (*n* = 100) for the OPLS-DA model corresponding to y-axis intercepts: R^2^ = (0.0, 0,302); Y^2^ = (0.0, −0,444). R^2^X = 0.783, Q^2^ = 0.177.

To determine the distinction between samples collected after 1 week, a second score plot was generated (Fig. 2a). Treated (blue dots) samples clustered together, clustering to the right. To further validate the OPLS-DA model, the 100 permutation tests was performed (Fig. 2b). The OPLS-DA model showed a degree of clustering with a goodness-of-fit (R^2^X = 0.783) with a predictability (Q^2^ = 0.177) as treated samples collected after 1 week gradually clustered to the right (Fig. 2b).

**Fig. 3:**
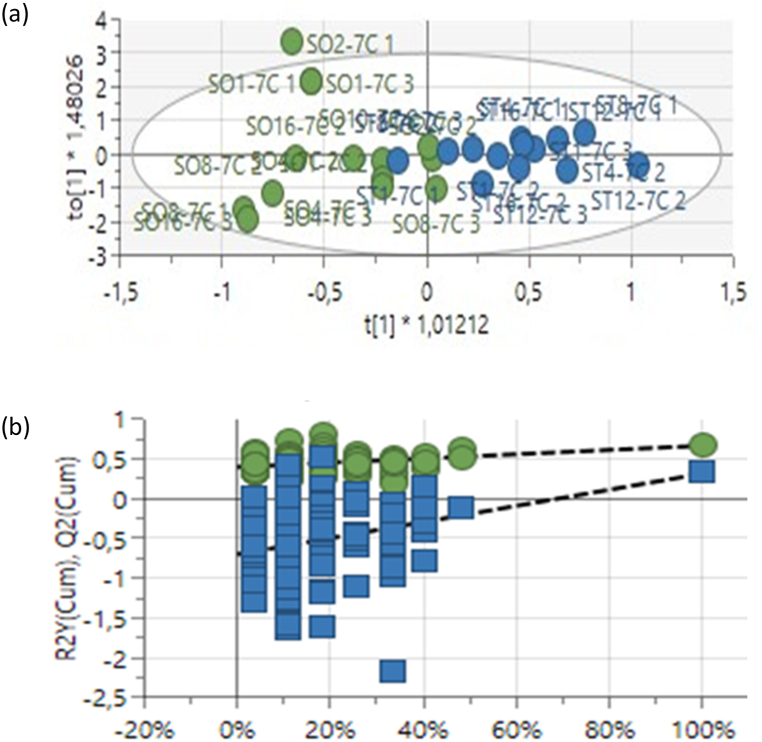
(a) OPLS-DA results of treated and untreated *Glycinemax* L. leaf extracts (1–3 weeks). Blue = treated, green = untreated. (b) The 100-permutation test used to validate the model (R^2^X = 0.795, R^2^Y = 0.611, Q^2^ = 0.408).

The OPLS-DA score plot for samples harvested 3 weeks after Photon treatment was generated (Fig. 3a). The statistical model revealed a distinct separation between treated and non-treated samples of *G. max*. Furthermore, the model showed a goodness-of-fit and predictability (R^2^X = 0.795, R^2^Y = 0.611 and Q^2^ = 0.408). To validate the model, 100 permutation tests were created (Fig. 3b).

Furthermore, the chemical shifts observed from ^1^H NMR metabolomic analysis were compared to those of standard compounds in Chenomx (Version 9.0) and the HMDB. Table 1 indicates compounds that were identified in *Glycine* *max* L. methanolic (D_2_O) extracts. Eight compounds were annotated in treated and untreated leaf samples collected after Photon application. Fumarate was significantly increased and visible at 12 h up to 3 weeks in treated samples, with GABA, alanine, asparagine, citrate and malate also in a higher concentration at certain collections in the treated samples.

**Table 1:** The chemical shifts and metabolite assignments of *Glycinemax* L. methanolic extracts that were in the leaf extracts.

Compound	1H NMR Chemical Shifts (ppm)	Chenomx (ppm)	Human Metabolite Database	Reference Chemical Shift (ppm)	Literature
aFumarate	6.5	6.5	6.51	6.51	[13,14]
aGamma-aminobutyric acid (GABA)	1.87 2.27 2.98	1.9 2.3 3.0	1.90 2.30 3.02	1.87 1.90 2.27 2.30 2.98 3.02	
aAlanine	1.45 1.49	1.5	1.47	1.47	[15]
aAsparagine	2.79 2.92	2.92		2.85 3.77	[16]
aCitrate	2.52 2.72	2.5 2.7	2.51 2.67	2.52 2.72	[16]
aMalate	2.64	2.4 2.64 2.66	2.38	2.66	[14]
Formate	8.45	8.40	8.49	8.46	[14]
Succinate	2.49	2.4	2.4	2.45	[17]
Cystathionine	2.75 3.80	2.1 2.2 2.7 3.1 3.8 3.9	2.22 2.72 3.85 3.95	2.23 2.74 3.86 3.96	[18]

a High after treatment.

**Fig. 4:**
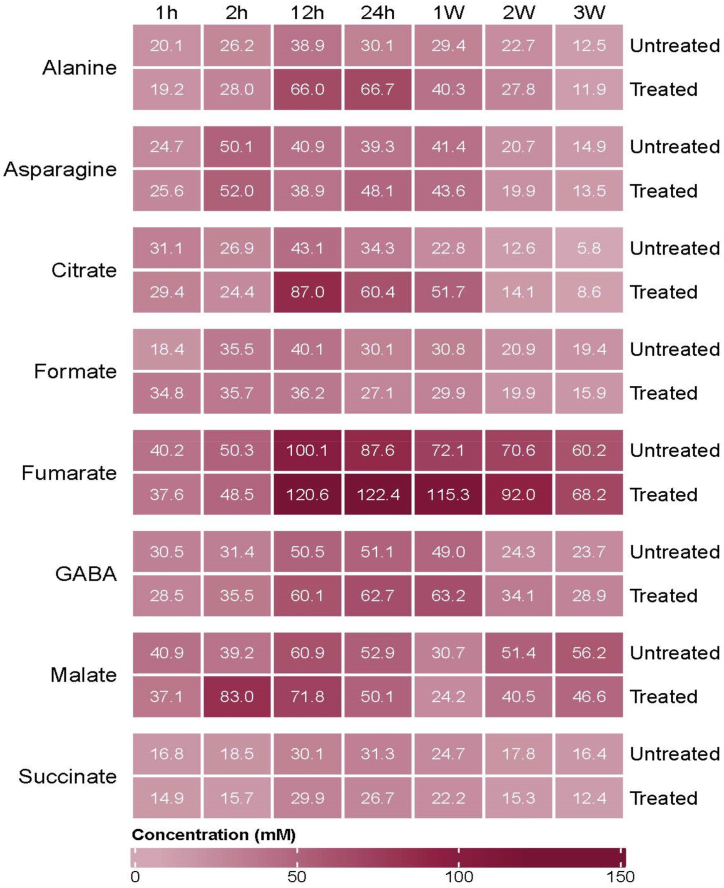
Various metabolites produced in response to dicarboxylic acids (Photon) application. A heat map displaying the concentrations of annotated metabolites present in treated and untreated *Glycinemax* L. leaves.

**Fig. 5:**
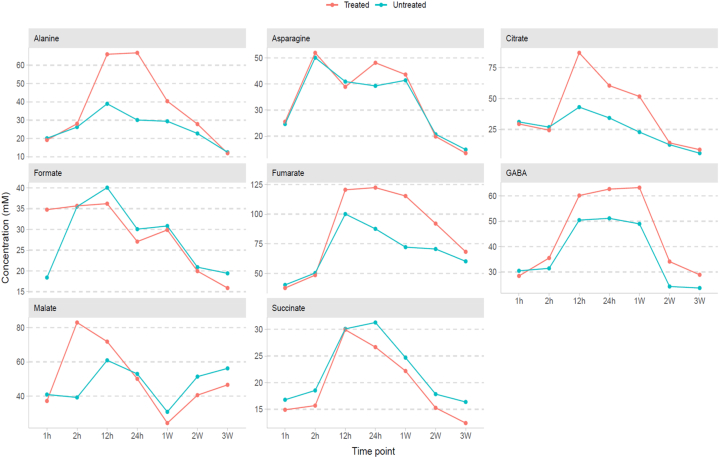
Changes in concentrations of metabolites upon photon treatment. Orange = treated; green = untreated.

The heatmap (Fig. 4) illustrates the concentrations of the identified metabolites that exhibited a rise from 1 h to 3 weeks after Photon treatment. Each detected metabolite's concentrations at various time points were determined by chemonx software that was used for annotation of metabolites. Furthermore, the graphs clearly depict the changes in the concentration of metabolites that were upregulated after the application of photon on *G*. max leaf extracts, with fumarate exhibiting the most elevated concentration (Fig. 5). The concentrations were calculated using chenomx software (NMR suite, version 9.0) using the TSP (0.01 %) as reference.

### 3.2 GC-MS

The GC-MS results confirmed the presence of the compounds as fatty acids methyl esters from the *Glycine* *max* L. methanol extracts, as shown in Table 2. The fatty acids that were detected in the leaf extracts were determined based on their molecular formula, molecular weight, retention time (RT) and area % (Table 2). Among the compounds that were identified are 11-octadecenoic acid, methyl ester; hexadecanoic acid, 15-methyl-, methyl ester; 9,12-octadecadienoic acid, methyl ester; octanoic acid, 2-dimethylaminoethyl ester; tridecanoic acid, methyl ester and fumaric acid, 2-dimethylaminoethyl octadecyl ester found in both treated and untreated samples collected up to 3 weeks following treatment.

**Table 2:** Fatty acids methyl esters revealed by GC-MS analysis of the methanol leaf extracts of *Glycinemax* L.

Name of Compound	Molecular Formula	Molecular Weight	Area %	Retention Time
7-Hexadecenoic acid, methyl ester (12h)	C_17_H_32_O_2_	268	2.33	17:34,9
Tridecanoic acid, methyl ester	C_14_H_28_O_2_	228	0.23	17:37,0
9,12-Octadecadienoic acid, methyl ester	C_19_H_34_O_2_	294	0.25	19:15,4
9-Octadecenoic acid (Z)-, methyl ester	C_19_H_36_O_2_	296	0.12	19:18,8
9,12,15-Octadecatrienoic acid, methyl ester, (Z,Z,Z).	C_19_H_32_O_2_	292	0.04	19:18,9
11-Octadecenoic acid, methyl ester Oleic acid methyl ester	C_19_H_36_O_2_	296	0.23	19:19,1
9,12,15-Octadecatrien-1-ol, (Z,Z,Z)-	C_18_H_32_O	264	0.15	19:19,3
Fumaric acid, 2-dimethylaminoethyl octadecyl ester	C_26_H_49_NO_4_	439	0.5	22:25,7
Dodecanoic acid, methyl ester	C_13_H_26_O_2_	214	0.38	19:31,4
Octanoic acid, methyl ester	C_12_H_25_NO_2_	215	1.32	22:26,4

### 3.3 Proteins detected in *Glycinemax* L. leaf samples

**Fig. 6:**
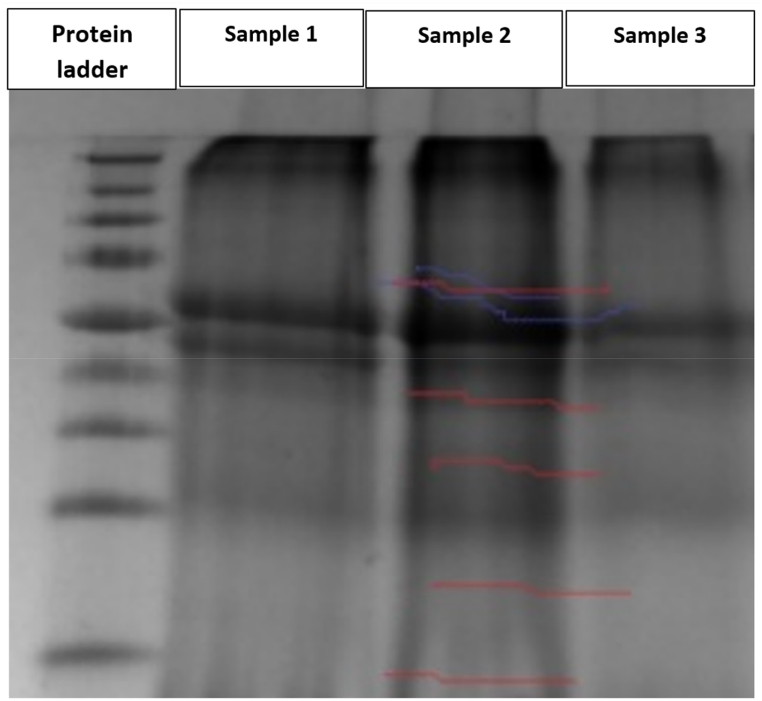
Sodium dodecyl sulfate-polyacrylamide gel electrophoresis image of proteins extracted from *Glycinemax* L. leaf samples. **Sample 1** = 12 h (treated), **Sample 2** = 3 weeks(treated), **Sample 3** = Control (untreated).

A one-dimensional gel electrophoresis system, coupled with MS application for the identification of proteins, was applied. Soybean leaf proteins were analyzed up to 3 weeks after spraying with Photon. Five bands each were prepared for the 3 lanes. Each lane (5 samples) was searched separately. The measured samples were collected 12 h, 1 week and 3 weeks after Photon treatment. A total of 274 proteins were identified in this study from Photon-treated and untreated samples (12 h, 1 week and 3 weeks) (Supplementary Material Table 1). Approximately 80 of the 274 extracted proteins were identical across all samples, with some slight differences on peptides (95 %) and % amino acid sequence coverage. The SDS-PAGE of proteins extracted from the *Glycine* *max* L. is shown in Fig. 6.

## 4 Discussion

### 4.1 ^1^H NMR analysis

Soybean (*Glycine* *max* L.) is one of the most researched legumes on a metabolic level, and several metabolites have been identified. Analyses were conducted on methanol leaf extracts obtained at various time intervals after treatment with a combination of dicarboxylic acids. The NMR-based metabolomic analysis conducted in this study revealed the existence of metabolites that were significantly increased in treated leaf extracts compared to the controls. The Photon-treated and untreated extracts obtained after 1 h, 2 h, and 12 h were not clearly distinguished by the supervised OPLS-DA. After conducting additional analysis with metabolite annotation, fumarate was significantly elevated in treated samples compared to untreated controls. Metabolites from a variety of classes were found in both treated and untreated *G*. max leaves. These included intermediates in the tricarboxylic acid (TCA) cycle (fumarate, formate, malate, succinate and citrate) and amino acids (4-aminobutyrate, alanine and asparagine). Most of these metabolites presented higher concentrations in the Photon-treated samples.

Interestingly, fumarate was found to be significantly elevated after 12 h up to 3 weeks of Photon treatment. This compound is known as an important component of the basic metabolism of plants. It has been reported that fumarate activates the Keap1-NRF2 metabolic pathway that is involved in the antioxidant defense mechanism [19,20]. Furthermore, NRF2 is the transcription factor that controls the production of antioxidant enzymes. The Keap1-NRF2 signaling pathway plays a critical role in modulating the adaptive cellular response to oxidative substances [21]. Additionally, fumarate is a component of the tricarboxylic acid cycle that can be digested to provide energy and carbon skeletons which can be used to produce other compounds [22]. Previous studies revealed changes in the fumaric acid concentration in *Arabidopsis* leaves using GC-MS [22]. According to the findings, fumaric acid concentrations in *Arabidopsis* leaves increased with plant age and light intensity. This agrees with the findings of this study, whereby the concentrations of fumarate in Photon-treated samples collected at midday (12 h, 24 h) were significantly increased. However [23], reported that fumarate levels significantly decreased on both short days and long days in *Arabidopsis* plants. Furthermore [24], found fumarate, succinate and other TCA cycle metabolites to be significantly down-regulated by NaCl treatment in *Aeluropus lagopoides* shoots. With a pathway analysis on the compounds fumarate, alanine and asparagine the alanine, aspartate and glutamate metabolic pathway as well as the central carbon metabolism in cancer metabolic pathway were provided as possible metabolic pathways, supporting the proposed involvement of fumarate in plant metabolism and Keap1-NRF2. The pathways are provided in the supplementary material as Fig. 1, Fig. 2.

In the treated samples, the increase in malate was only detectable after 12 h, and it continued to decline for 3 weeks. Along with fumarate, malate is an intermediary in the TCA cycle. In a comprehensive metabolic profiling study conducted by Ref. [25], malate and fumarate were detected. The results revealed that the levels of these metabolites exhibited diurnal fluctuations, rising during the day and falling at night. Furthermore, the study suggests that both fumarate and malate act as carbon storage compounds that can be degraded in *Arabidopsis* under stress conditions [26]. discovered that TCA cycle metabolites such as citric acid, ketoglutaric acid, fumaric acid and malic acid reduced in the primary root of maize plants under drought stress. However [27], reported that *Arabidopsis* leaves deprived of Gibberellins increased malate and fumarate levels 1.5-fold in ambient [CO_2_] compared to high [CO_2_].

Other compounds detected include GABA, asparagine and alanine - all of which have been linked with amino acid metabolism and stress tolerance in plants. The metabolic responses of these compounds to both biotic and abiotic stresses are well documented. Amino acids such as alanine, GABA, glutamate and asparagine increased in maize plants subjected to salinity stress [28]. These findings are consistent with those of [24], who studied the reaction of the hylophytic species *Aelurropus lagopoides* to salt stress and found that metabolites related to amino acid biosynthesis such as alanine, asparagine, and other amino acids were up-regulated. In contrast, several investigations observed a reduction in amino acids during salt stress (29). The increase in amino acids observed in the current study may indicate that they play a role in the adaptive response of soybean plants following Photon treatment.

### 4.2 GC-MS analysis

The Fatty acid methyl esters are analyzed by GC-MS to determine the fatty acid composition in organisms [29]. In this study, the GC-MS approach was used to confirm the fatty acids present in methanolic extracts. The GC-MS examination of *Glycine* max leaf extracts revealed the presence of 10 fatty acid methyl esters as these are precursors in the jasmonic acid pathway [22]. utilized GC-MS to investigate the fatty acid composition of *Arabidopsis* leaf tissue. In this study, it is noteworthy that methyl esters of fatty acids, including α-linolenic acid, oleic acid, and linolenic acid, were identified and subsequently reported as 9,12,15-Octadecatrienoic acid, (Z,Z,Z)-, 9-Octadecenoic acid (Z)- and 9,12,15-Octadecatrien-1-ol, (Z,Z,Z)-. Linolenic acid and oleic acid were present in most samples from 1 h to 3 weeks of Photon treatment. These unsaturated fatty acids are prevalent in most plant species because they modulate stress signaling and serve as precursors to other bioactive compounds, such as jasmonates [30]. Jasmonic acid is known to be synthesized from linolenic acid via the octadecanoic acid pathway. An extensive review on the synthesis and signaling of jasmonic acid in response to abiotic stressors was reported. According to this review, jasmonic acid has been shown to increase the plant's resistance to abiotic stresses by stimulating the production of antioxidant enzymes and other defense compounds [31], and it seems that several proteins are involved in the signaling pathways.

### 4.3 Proteomics analysis

The approach used detected a number of proteins in *G*. max leaf extracts. Among the 274 proteins detected in soybean leaf extracts, some stress proteins, with potential significant functions under diverse stress conditions, were found in the Photon-treated samples, while they were absent in the control group. These proteins include ferritins, chalcone--flavanone isomerase 1A, ferredoxin-thioredoxin reductase catalytic chain and calmodulin-2.

Ferritin 1, 2 and 4 were discovered in treated extracts but not in the untreated sample. Ferritin is the iron store in plants that plays a vital function during development and under stress conditions, preventing iron-induced oxidative stress in the plant [32]. Plant ferritins of various food crops were characterized in previous studies. Ferritin in leaves may also function as a precursor for the formation of iron-containing proteins. This protein was previously reported in *Pisum sativum* [33] and *G. max* [34]. In soybean [35], found ferritin in seeds with subunits (H-1) and (H-2), which may perform cooperative roles in the release of iron atoms. The presence of ferritin may help to protect plants from oxidative stress, which can be caused by iron redundance [36].

It has been reported that chalcone-isomerase increases the transcript abundance of structural genes in the flavonoids biosynthesis pathway, resulting in increased flavonoids production under abiotic stressors in transgenic plants [37]. This enzyme is also important in response to biotic stimuli such as pathogens. Overexpression of chalcone-isomerase (CHI1A) was reported to increase Phytophthora sojae resistance in soybean roots [38].

Calmodulin (CaM) is one of the calcium-binding proteins that plays an important role in plants during environmental stresses. It acts as a calcium ion (Ca^2^+) signal transducer in the plant stress signaling pathway [39]. In addition, it is implicated in the calcium signal transduction pathway by altering its connections with target proteins including kinases and phosphatases [40]. CaMs have been widely investigated in plant responses to abiotic stressors. In a study conducted by Ref. [39], it was found that CaM activity increased metabolic enzymes in the energy pathway during salt stress in rice, maintaining energy production under photosynthesis limitation. Another study demonstrated the involvement of CaM in heat shock signal transduction in *Arabidopsis*. CaM overexpression significantly improved heat tolerance in both the AtCaM3 knockout and the wild-type background [32].

According to our findings, heat shock proteins (HSPs) were found in both treated and untreated samples. It is well established that HSPs play a critical role in conferring abiotic stress tolerance [6]. Compared to other stresses, heat shock proteins are well-known for their protection against heat stress [41]. alluded that the heat stress tolerance in soybean plants was linked to enhanced HSP genes. The presence of HSPs in the present study may play an active role in the stress response of soybeans, particularly under heat stress. As a result, further analysis is required to understand the response mechanism of these genes under heat stress. Moreover, it has been reported that HSPs maintain cellular homeostasis in plants during drought adaptation and under regular plant life conditions [42]. A study conducted by Ref. [43] investigated HSP70 function in *Nicotiana tabacum*. The findings revealed that tobacco seedlings subjected to drought stress overexpressed NtHSP70. In addition, there was an increase in plant growth, suggesting that HSP70 is involved in drought tolerance in plants. While exceptions do exist, overall improvement in stress tolerance seems to be the consequence of increased HSPs in various plant species. To confirm changes in HSPs expression, photon-treated soybeans subjected to different stressors will be required.

The results of this study showed that after a week of Photon treatment, ferredoxin-thioredoxin was present in the Photon-treated *G*. max leaf samples. This protein was detected in the treated samples but not in the untreated samples. Under physiological and stress conditions, ferredoxin-thioredoxin reductase is reported to remove excess reducing power and prevents stroma overreduction [44]. In a comprehensive review done by Ref. [45], it was revealed that in low-light conditions, nitrogen regulatory protein C controls the regulation of the Calvin cycle enzymes by interacting with the Fd/FTR/Trx system. According to the findings of the current study, several of these enzymes including Fructose 1.6 bisphosphatase (FBPase), ATP synthase, malate dehydrogenase and RuBisCo-associated protein, were present in both Photon-treated and control samples. Interestingly, five ATP synthase-related proteins were present in all the samples. In a previous study conducted by Ref. [46], three ATP synthase-related proteins that were significantly expressed in heat-sensitive chrysanthemum were found to be downregulated in response to heat stress, indicating that ATP synthase may play a role in adaptation to heat stress. In addition, the quantity of FBPase was shown to be greater in heat-tolerant varieties than in heat-sensitive varieties. The proteins discovered in the treated samples, namely calmodulin, chalcone isomerase, and ferredoxin thioredoxin reductase, are all involved in the metabolism of iron sequestration and transport, as determined by the GO enrichment study (https://geneontology.org/).

## 5 Conclusion

With continuously created priming agents, numerous research can be conducted to determine the method of action in plants. In this study, NMR-based metabolomics was used to detect metabolites of soybean leaf extracts after the application of Photon. Leaf samples were taken at various time intervals (1 h, 2 h, 12 h, 24 h, 1 week, 2 weeks and 3 weeks) after application. OPLS-DA was used for class separations between treated and untreated samples. Several compounds, including organic acids, were enhanced in Photon-treated plants, with fumarate being the most prominent. The GC-MS analysis also confirmed the fatty acids that are precursors of jasmonic acid. Further analysis was conducted to detect proteins involved in plant stress tolerance. Extracts from soybean leaves treated with Photon contained proteins such as ferritins, CaM, and Chalcone-flavanone isomerase 1A, whereas untreated extracts did not. The activities of these proteins regulate the response of plants to abiotic stresses to varied degrees, and further analysis should be undertaken to fully understand how they respond to Photon. This study for the first time documents the effect of dicarboxylic acids (Photon) on soybean leaves using NMR-based metabolomics and proteomics analysis. These techniques were successful in highlighting chemicals and proteins when applied. However, more research with further analysis is needed to determine the effects of Photon on each of these proteins to fully comprehend their response. In conclusion, we suggest that elevated quantities of fumarate could be related to the Keap1-NRF2 metabolic pathway. NMR-based metabolomics and proteomics proved to be suitable techniques to identify metabolic changes in soybean but can be applied with equal success to other crops as well. The study provides the first report on the metabolic alteration on soybean to the application of a priming agent (photon). The metabolomic and proteomic analysis provides an overview of how soybean prepares to mitigate possible stress stimuli in the environment.

## Data availability statement

The data was uploaded in Mendeley, a publicly available database. The citation to the database is: Msomi, Mhlonipheni (2024), “Proteomics analysis of *Glycine* max leaf extracts”, Mendeley Data, V2, https://doi.10.17632/fzygvnzbw7.2.

## CRediT authorship contribution statement

**Mhlonipheni Nhlakanipho Msomi:** Writing – original draft, Visualization, Validation, Project administration, Methodology, Investigation, Formal analysis, Data curation, Conceptualization. **Gerhard Prinsloo:** Writing – original draft, Visualization, Validation, Project administration, Methodology, Investigation, Formal analysis, Data curation, Conceptualization. **Noluyolo Nogemane:** Writing – original draft, Visualization, Validation, Project administration, Methodology, Formal analysis, Data curation, Conceptualization.

## Declaration of competing interest

The authors declare that they have no known competing financial interests or personal relationships that could have appeared to influence the work reported in this paper.

## References

[1] Wiszniewska A. (2021). Priming strategies for benefiting plant performance under toxic trace metal exposure. Plants.

[2] Benkeblia N., Shinano T., Osaki M. (2007). Metabolite profiling and assessment of metabolome compartmentation of soybean leaves using non-aqueous fractionation and GC-MS analysis. Metabolomics.

[3] Krasensky J., Jonak C. (2012). Drought, salt, and temperature stress-induced metabolic rearrangements and regulatory networks. J. Exp. Bot..

[4] Feng Z., Ding C., Li W., Wang D., Cui D. (2020). Applications of metabolomics in the research of soybean plant under abiotic stress. Food Chem..

[5] Yamaguchi-Shinozaki K., Shinozaki K. (2006). Transcriptional regulatory networks in cellular responses and tolerance to dehydration and cold stresses. Annu. Rev. Plant Biol..

[6] Mishra R., Pandey I.K., Khan M.I.R., Reddy P.S., Gupta R. (2022). Metabolites and Abiotic Stress Tolerance in Plants.

[7] Das A., Rushton P.J., Rohila J.S. (2017). Metabolomic profiling of soybeans (*Glycine max* L.) reveals the importance of sugar and nitrogen metabolism under drought and heat stress. Plants.

[8] Holmes M.V., Millwood I.Y., Kartsonaki C., Hill M.R., Bennett D.A., Boxall R., Guo Y., Xu X., Bian Z., Hu R., Walters R.G., Chen J., Ala-Korpela M., Parish S., Clarke R.J., Peto R., Collins R., Li L., Chen Z., China Kadoorie Biobank Collaborative Group (2018). Lipids, lipoproteins, and metabolites and risk of myocardial infarction and stroke. J. Am. Coll. Cardiol..

[9] Mediani A., Abas F., Khatib A., Maulidiani H., Shaari K., Choi Y.H., Lajis N.H. (2012). ^1^H-NMR-based metabolomics approach to understanding the drying effects on the phytochemicals in *Cosmos caudatus*. Food Res. Int..

[10] Sermakkani M., Thangapandian V. (2012). GC-MS analysis of *Cassia Italica* leaf methanol extract. Asian J Pharm Clin Res.

[11] Walker J.M., Walker J.M. (2009). The Protein Protocols Handbook.

[12] Sobhanian H., Motamed N., Jazii F.R., Nakamura T., Komatsu S. (2010). Salt stress induced differential proteome and metabolome response in the shoots of *Aeluropus lagopoides* (Poacea), a halophyte C (4) plant. J. Proteome Res..

[13] Akhtar M.T., Samar M., Shami A.A., Mumtaz M.W., Mukhtar H., Tahir A., Shahzad-ul-Hussan S., Chaudhary S.U., Kaka U. (2021). ^1^H-NMR-Based Metabolomics: an integrated approach for the detection of the adulteration in chicken, chevon, beef and donkey meat. Molecules.

[14] Oh J., Yoon D., Han J., Choi H., Sung G. (2018). ^1^H NMR based metabolite profiling for optimizing the ethanol extraction of *Wolfiporia cocos*. Saudi J. Biol. Sci..

[15] Casadei-Gardini A., Del Coco L., Marisi G., Conti F., Rovesti G., Ulivi P., Canale M., Frassineti G.L., Foschi F.G., Longo S., Fanizzi F.P., Giudetti A. (2020). ^1^H-NMR Based serum metabolomics highlights different specific biomarkers between early and advanced hepatocellular carcinoma stages. Cancers.

[16] Sundekilde U.K., Larsen L.B., Bertram H.C. (2013). NMR-based milk metabolomics. Metabolites.

[17] Gao F., Chao J., Guo J., Zhao L., Tian H. (2021). ^1^H NMR-based metabolomics to identify resistance-related metabolites in *Astragalus membranaceus* var. *mongholicus* against *Fusarium* root rot. Intl J Agric Biol.

[18] Branzoli F., Deelchand D.K., Sanson M., Lehéricy S., Marjańska M. (2019). In vivo 1 H MRS detection of cystathionine in human brain tumors. Magn. Reson. Med..

[19] Wu W.L., Papagiannakopoulos T. (2020). The pleiotropic role of the KEAP1/NRF2 pathway in cancer. Annu. Rev. Cancer Biol..

[20] Lee S., Hu L. (2020). Nrf2 activation through the inhibition of Keap1-Nrf2 protein-protein interaction. Med. Chem. Res..

[21] Liu S., Pi J., Zhang Q. (2022). Signal amplification in the KEAP1-NRF2-ARE antioxidant response pathway. Redox Biol..

[22] Chia D.W., Yoder T.J., Reiter W.D., Gibson S.I. (2000). Fumaric acid: an overlooked form of fixed carbon in *Arabidopsis* and other plant species. Planta.

[23] Zell M.B., Fahnenstich H., Maier A., Saigo M., Voznesenskaya E.V., Edwards G.E., Andreo C., Schleifenbaum F., Zell C., Drincovich M.F., Maurino V.G. (2010). Analysis of *Arabidopsis* with highly reduced levels of malate and fumarate sheds light on the role of these organic acids as storage carbon molecules. Plant Physiol.

[24] Takuji N., Keiki O., Noureddine B., Jun W., Toshihiro W., Hideyuki M., Hirofumi U., Setsuko K., Takuro S., Bilyeu K., Ratnaparkhe M.B., Kole C. (2010). Metabolomics Approach in Soybean.

[25] Fahnenstich H., Saigo M., Niessen M., Zanor M.I., Andreo C.S., Fernie A.R., Drincovich M.F., Flu gge U.I., Maurino V.G. (2007). Alteration of organic acid metabolism in *A. thaliana* overexpressing the maize C_4_-NADP malic enzyme causes accelerated senescence during extended darkness. Plant Physiol.

[26] Li P., Yang X., Wang H., Pan T., Yang J., Wang Y., Xu Y., Yang Z., Xu C. (2021). Metabolic responses to combined water deficit and salt stress in maize primary roots. J. Integr. Agric..

[27] Ribeiro D.M., Araujo W.L., Fernie A.R., Schippers J.H., Mueller-Roeber B. (2012). Action of gibberellins on growth and metabolism of Arabidopsis plants associated with high concentration of carbon dioxide. Plant Physiol.

[28] Gavaghan C.L., Li J.V., Hadfield S.T., Hole S., Nicholson J.K., Wilson I.D., Howe P.W.A., Stanley P.D., Holmes E. (2011). Application of NMR-based metabolomics to the investigation of salt stress in maize (*Zea mays* L.). Phytochem. Anal..

[29] Carrapiso A.I., Garcia C. (2000). Development in lipid analysis: some new extraction techniques and in situ transesterifi cation. Lipids.

[30] He M., Ding N. (2020). Plant unsaturated fatty acids: multiple roles in stress response. Front. Plant Sci..

[31] Ali M.S., Baek K.H. (2020). Jasmonic acid signaling pathway in response to abiotic stresses in plants. Int. J. Mol. Sci..

[32] Zhang W., Zhou R.G., Gao Y.J., Zheng S.Z., Xu P., Zhang S.Q., Sun D.Y. (2009). Molecular and genetic evidence for the key role of AtCaM3 in heat-shock signal transduction in *Arabidopsis*. Plant Physiol.

[33] Mediani A., Abas F., Khatib A., Maulidiani H., Shaari K., Choi Y.H., Lajis N.H. (2012). ^1^H-NMR-based metabolomics approach to understanding the drying effects on the phytochemicals in *Cosmos caudatus*. Food Res. Int..

[34] Ravet K., Touraine B., Kim S.A., Cellier F., Thomine S., Guerinot M.L., Briat J.F., Gaymard F. (2009). Post-translational regulation of AtFER2 ferritin in response to intracellular iron trafficking during fruit development in *Arabidopsis*. Mol. Plant.

[35] Masuda T., Goto F., Yoshihara T. (2001). A novel plant ferritin subunit from soybean that is related to a mechanism in iron release. J. Biol. Chem..

[36] Yadav K., Patel P., Srivastava A.K.G., Ganapathi T.R. (2017). Overexpression of native ferritin gene MusaFer1 enhances iron content and oxidative stress tolerance in transgenic banana plants. PLoS One.

[37] Jayaraman K., Venkat K., Mithra S.A., Sivakumar S.R., Gayatri T., Visawanathan C., Trilochan M., Pranab M. (2021). Stress-inducible expression of chalcone isomerase2 gene improves accumulation of flavonoids and imparts enhanced abiotic stress tolerance to rice. Environ. Exp. Bot..

[38] Zhou Y., Huang J.l., Zhang X.l., Zhu L., Wang X.F., Guo N., Zhao J.M., Xing H. (2018). Overexpression of chalcone isomerase (CHI) increases resistance against *Phytophthora sojae* in soybean. J. Plant Biol..

[39] Yuenyong W., Chinpongpanich A., Comai L., Chadchawan S., Buaboocha T. (2018). Downstream components of the calmodulin signaling pathway in the rice salt stress response revealed by transcriptome profiling and target identification. BMC Plant Biol..

[40] Briat J.F., Duc C., Ravet K., Gaymard F. (2010 Aug). Ferritins and iron storage in plants. Biochim. Biophys. Acta.

[41] Song K., Yim W.C., B Lee (2017). Expression of heat shock proteins by heat stress in soybean. Plant Breed Biotech.

[42] Aghaie P., Tafreshi S.A.H. (2020). Central role of 70-kDa heat shock protein in adaptation of plants to drought stress. Cell Stress and Chaperones.

[43] Cho E.K., Choi Y.J. (2009). A nuclear-localized HSP70 confers thermoprotective activity and drought-stress tolerance on plants. Biotechnol. Lett..

[44] Eckardt N.A. (2006). Ferredoxin-thioredoxin system plays a key role in plant response to oxidative stress. Plant Cell.

[45] Kang Z., Qin T., Zhao Z. (2019). Thioredoxin and thioredoxin reductase in chloroplasts: a review. Gene.

[46] Zhang Y., Sun M., Zhang Q. (2014). Proteomic analysis of the heat stress response in leaves of two contrasting chrysanthemum varieties. Plant Omics J.

