# Supplementary material for: 1H-NMR-based metabolomic profiling and proteomic analysis of soybean (Glycine max L.) in response to dicarboxylic acids (photon) application as a stress priming agent
Source: Heliyon. 2024 Sep 7;10(18):e37466. doi: 10.1016/j.heliyon.2024.e37466 (PMC11414495; doi:10.1016/j.heliyon.2024.e37466)
Supplement: Multimedia component 1 [file mmc1.docx]

**Supplementary material**

**Table 1:** The list of protein identification-false discovery rates generated from Photon-treated *Glycine max* L. leaf extracts. **Total score**: Measurement of all peptide evidence for a protein (not quantitative). **%Cov(95):** % amino acid sequence coverage when using peptides identified at 95% confidence (those that are included in the analysis). **Peptides (95):** the number of unique peptides used as evidence for the protein hit.

| **Total** | **Accession no** | **Name** | **%Cov(95)** | **Peptides**  **(95%)** |
| --- | --- | --- | --- | --- |
| 70,1 | sp\|P38417\|LOX4 | Linoleate 9S-lipoxygenase-4 | 50,29000044 | 40 |
| 61,44 | sp\|P27066\|RBL | Ribulose bisphosphate carboxylase large chain | 61,26000285 | 59 |
| 58,37 | sp\|Q2PMV0\|ATPB | ATP synthase subunit beta, chloroplastic | 83,93999934 | 50 |
| 44,28 | sp\|P24095\|LOXX | Seed linoleate 9S-lipoxygenase | 33,21999907 | 23 |
| 40,43 | sp\|Q2PMS8\|ATPA | ATP synthase subunit alpha, chloroplastic | 57,84000158 | 29 |
| 29,46 | sp\|I1K0K6\|EFGC2 | Elongation factor G-2, chloroplastic | 26,03000104 | 16 |
| 29,45 | sp\|P15490\|VSPA | Stem 28 kDa glycoprotein | 63,77999783 | 24 |
| 29,23 | sp\|Q43467\|EFTU1 | Elongation factor Tu, chloroplastic | 46,34999931 | 16 |
| 27,8 | sp\|P10538\|AMYB | Beta-amylase | 33,27000141 | 14 |
| 29,09 | sp\|Q01915\|ATPAM | ATP synthase subunit alpha, mitochondrial | 34,83999968 | 17 |
| 24,67 | sp\|Q03773\|E13A | Glucan endo-1,3-beta-glucosidase | 50,72000027 | 12 |
| 24 | sp\|Q2PMQ9\|PSBB | Photosystem II CP47 reaction center protein | 29,71999943 | 15 |
| 23,92 | sp\|P49161\|CYF | Cytochrome f | 51,88000202 | 13 |
| 22,02 | sp\|P00865\|RBS1 | Ribulose bisphosphate carboxylase small subunit, chloroplastic 1 | 56,73999786 | 17 |
| 21,07 | sp\|P10743\|VSPB | Stem 31 kDa glycoprotein | 50 | 11 |
| 20,04 | sp\|Q2PMT9\|PSBC | Photosystem II CP43 reaction center protein | 26,85000002 | 14 |
| 20 | sp\|P09756\|CB23 | Chlorophyll a-b binding protein 3, chloroplastic | 72,61999846 | 15 |
| 20 | sp\|Q2PMU9\|ATPE | ATP synthase epsilon chain, chloroplastic | 84,32999849 | 17 |
| 18,99 | sp\|P25698\|EF1A | Elongation factor 1-alpha | 24,37999994 | 10 |
| 18,02 | sp\|P28759\|SODF | , | 39,10999894 | 12 |
| 17,8 | sp\|O48561\|CATA4 | Catalase-4 | 26,21999979 | 10 |
| 17,63 | sp\|P39657\|RUAP | RuBisCO-associated protein | 46,29000127 | 9 |
| 17,03 | sp\|Q2PMN8\|NDHH | NAD(P)H-quinone oxidoreductase subunit H, chloroplastic | 29,51999903 | 9 |
| 16,12 | sp\|P29136\|MEP1 | Metalloendoproteinase 1 | 28,51999998 | 8 |
| 15,85 | sp\|P93164\|GGH | Gamma-glutamyl hydrolase | 26,89999938 | 8 |
| 15,6 | sp\|Q2PMT8\|PSBD | Photosystem II D2 protein | 28,90000045 | 11 |
| **14,88** | **sp\|P26413\|HSP70** | **Heat shock 70 kDa protein** | **19,38000023** | **9** |
| 14,22 | sp\|Q2PMS9\|ATPF | ATP synthase subunit b, chloroplastic | 40,5400008 | 8 |
| 14,03 | sp\|Q2PMU5\|RR4 | 30S ribosomal protein S4, chloroplastic | 37,81000078 | 7 |
| 14 | sp\|Q96450\|1433A | 14-3-3-like protein A | 34,24000144 | 8 |
| 13,83 | sp\|P24099\|GLNA1 | Glutamine synthetase cytosolic isozyme 1 | 25,35000145 | 7 |
| **13,65** | **sp\|Q42796\|F16P1** | **Fructose-1,6-bisphosphatase, chloroplastic** | **23,88000041** | **8** |
| 13,36 | sp\|P28551\|TBB3 | Tubulin beta chain (Fragment) | 22,55000025 | 7 |
| 12,37 | sp\|P54774\|CDC48 | Cell division cycle protein 48 homolog | 9,294000268 | 6 |
| 12,11 | sp\|P02957\|PSBA | Photosystem II protein D1 | 20,11000067 | 8 |
| 12 | sp\|Q39839\|NDK1 | Nucleoside diphosphate kinase 1 | 40,2700007 | 6 |
| 12,01 | sp\|Q02909\|CAPP1 | Phosphoenolpyruvate carboxylase, housekeeping isozyme | 8,27300027 | 7 |
| 11,59 | sp\|P31174\|NDHJ | NAD(P)H-quinone oxidoreductase subunit J, chloroplastic | 53,15999985 | 6 |
| 11,57 | sp\|P02581\|ACT1 | Actin-1 | 24,67000037 | 6 |
| 11,07 | sp\|O22518\|RSSA | 40S ribosomal protein SA | 26,44999921 | 7 |
| 10,78 | sp\|Q9M4T8\|PSA5 | Proteasome subunit alpha type-5 | 28,27000022 | 5 |
| 10,6 | sp\|Q9AWB2\|SIR | Sulfite reductase [ferredoxin], chloroplastic (Fragment) | 16,58000052 | 7 |
| 10,51 | sp\|Q06197\|IDHC | Isocitrate dehydrogenase [NADP] | 17,91999936 | 6 |
| 10,29 | sp\|P0CG89\|H4 | Histone H4 | 41,74999893 | 6 |
| 10,2 | sp\|P62302\|RS13 | 40S ribosomal protein S13 | 41,71999991 | 5 |
| 10,06 | sp\|P07135\|RR7 | 30S ribosomal protein S7, chloroplastic | 27,7399987 | 6 |
| **10,06** | **sp\|P19976\|FRI1** | **Ferritin-1, chloroplastic** | **20,0000003** | **6** |
| 10,02 | sp\|Q2PMU2\|PSAB | Photosystem I P700 chlorophyll a apoprotein A2 | 9,127999842 | 5 |
| 9,8 | sp\|P25096\|P21 | Protein P21 | 35,6400013 | 6 |
| 9,78 | sp\|Q39836\|GBLP | Guanine nucleotide-binding protein subunit beta-like protein | 23,69000018 | 5 |
| 9,41 | sp\|P69325\|UBIQP | Polyubiquitin | 61,64000034 | 5 |
| 9,02 | sp\|Q944T2\|TCTP | Translationally-controlled tumor protein homolog | 28,56999934 | 5 |
| 8,1 | sp\|P45621\|GSA | Glutamate-1-semialdehyde 2,1-aminomutase, chloroplastic | 16,74000025 | 5 |
| 8,06 | sp\|P17093\|RS11 | 40S ribosomal protein S11 | 25,78999996 | 4 |
| 8,01 | sp\|Q2PMP9\|RK14 | 50S ribosomal protein L14, chloroplastic | 40,16000032 | 6 |
| 7,87 | sp\|Q41219\|LEGRE | Leghemoglobin reductase | 13,5800004 | 4 |
| 7,62 | sp\|Q2PMQ0\|RR8 | 30S ribosomal protein S8, chloroplastic | 33,57999921 | 4 |
| 7,24 | sp\|Q2PMP7\|RR3 | 30S ribosomal protein S3, chloroplastic | 12,5 | 3 |
| **6,68** | **sp\|O49856\|FTRC** | **Ferredoxin-thioredoxin reductase catalytic chain, chloroplastic** | **20,13999969** | **4** |
| 6,41 | sp\|Q2PMP8\|RK16 | 50S ribosomal protein L16, chloroplastic | 31,11000061 | 3 |
| **8,31** | **sp\|Q94IC4\|FRI2** | **Ferritin-2, chloroplastic** | **20,22999972** | **5** |
| 6,22 | sp\|Q2PMU3\|PSAA | Photosystem I P700 chlorophyll a apoprotein A1 | 3,866999969 | 3 |
| 6,11 | sp\|Q2PMM3\|RK2B | 50S ribosomal protein L2-B, chloroplastic | 16,06000066 | 3 |
| 6,09 | sp\|P07134\|RR12 | 30S ribosomal protein S12, chloroplastic | 19,50999945 | 3 |
| 6,07 | sp\|P48640\|GSHRP | Glutathione reductase, chloroplastic | 8,455999941 | 3 |
| 6,03 | sp\|Q2PMR0\|CLPP | ATP-dependent Clp protease proteolytic subunit | 28,56999934 | 3 |
| **6,02** | **sp\|P62163\|CALM2** | **Calmodulin-2** | **27,52000093** | **3** |
| 6,01 | sp\|Q2PMN9\|RR15 | 30S ribosomal protein S15, chloroplastic | 32,22000003 | 3 |
| 6 | sp\|Q8W171\|CYP1 | Peptidyl-prolyl cis-trans isomerase 1 | 19,76999938 | 3 |
| 10,03 | sp\|Q96451\|1433B | 14-3-3-like protein B (Fragment) | 25,51000118 | 6 |
| 6,04 | sp\|C6SXE1\|CYNS | Cyanate hydratase | 18,79000068 | 3 |
| 13,68 | sp\|P12471\|CB21 | Chlorophyll a-b binding protein, chloroplastic (Fragment) | 53,06000113 | 10 |
| 5,37 | sp\|P50346\|RLA0 | 60S acidic ribosomal protein P0 | 18,12999994 | 3 |
| **5,53** | **sp\|Q93XE6\|CFI1A** | **Chalcone--flavanone isomerase 1A** | **25,22999942** | **4** |
| 5,34 | sp\|P35055\|HEM6 | Oxygen-dependent coproporphyrinogen-III oxidase, chloroplastic | 8,312000334 | 3 |
| 5,06 | sp\|O48551\|PSA6 | Proteasome subunit alpha type-6 | 19,11000013 | 3 |
| 4,75 | sp\|P46417\|GSTX3 | Glutathione S-transferase 3 | 9,589000046 | 4 |
| 4,59 | sp\|P43210\|HEM2 | Delta-aminolevulinic acid dehydratase, chloroplastic | 5,339999869 | 2 |
| 4,56 | sp\|Q2PMQ5\|CYB6 | Cytochrome b6 | 19,52999979 | 3 |
| 11,37 | sp\|P09186\|LOX3 | Seed linoleate 9S-lipoxygenase-3 | 8,05099979 | 6 |
| 4,35 | sp\|Q02226\|COXT | Cytochrome c oxidase subunit 2, mitochondrial (Fragment) | 9,391000122 | 3 |
| 4,19 | sp\|Q2PMN6\|NDHI | NAD(P)H-quinone oxidoreductase subunit I, chloroplastic | 23,3099997 | 3 |
| **8,09** | **sp\|Q948P6\|FRI3** | **Ferritin-3, chloroplastic** | **24,22000021** | **5** |
| 4,04 | sp\|P37228\|MDHG | Malate dehydrogenase, glyoxysomal | 7,365000248 | 2 |
| 4,03 | sp\|Q2PMU4\|YCF3 | Photosystem I assembly protein Ycf3 | 8,92900005 | 2 |
| 13,06 | sp\|P09439\|LOX2 | Seed linoleate 9S-lipoxygenase-2 | 7,745999843 | 7 |
| 4,01 | sp\|Q42806\|KPYC | Pyruvate kinase, cytosolic isozyme | 5,674999952 | 2 |
| 4,01 | sp\|P07816\|RR19 | 30S ribosomal protein S19, chloroplastic | 22,83000052 | 2 |
| 4,01 | sp\|Q2PMS5\|RR16 | 30S ribosomal protein S16, chloroplastic | 24,71999973 | 2 |
| 4,01 | sp\|Q2PMR2\|RR18 | 30S ribosomal protein S18, chloroplastic | 9,708999842 | 2 |
| 4 | sp\|Q2PMR7\|PSBE | Cytochrome b559 subunit alpha | 22,89000005 | 3 |
| 4 | sp\|Q2PMQ4\|PETD | Cytochrome b6-f complex subunit 4 | 10,00000015 | 2 |
| 4 | sp\|Q2PMQ2\|RR11 | 30S ribosomal protein S11, chloroplastic | 17,38999933 | 2 |
| 4 | sp\|Q2PMN3\|PSAC | Photosystem I iron-sulfur center | 27,1600008 | 2 |
| 4 | sp\|Q02243\|WIN | Wound-induced protein (Fragment) | 31,36999905 | 2 |
| 3,68 | sp\|Q43463\|RAB7 | Ras-related protein Rab7 | 10,18999964 | 2 |
| 3,63 | sp\|Q9ZS21\|LGUL | Lactoylglutathione lyase | 14,04999942 | 2 |
| 3,22 | sp\|Q2PMQ6\|PSBH | Photosystem II reaction center protein H | 23,28999937 | 2 |
| 3,2 | sp\|P31175\|NDHK | NAD(P)H-quinone oxidoreductase subunit K, chloroplastic | 11,7899999 | 2 |
| 3,12 | sp\|P32110\|GSTX6 | Probable glutathione S-transferase | 8,889000118 | 2 |


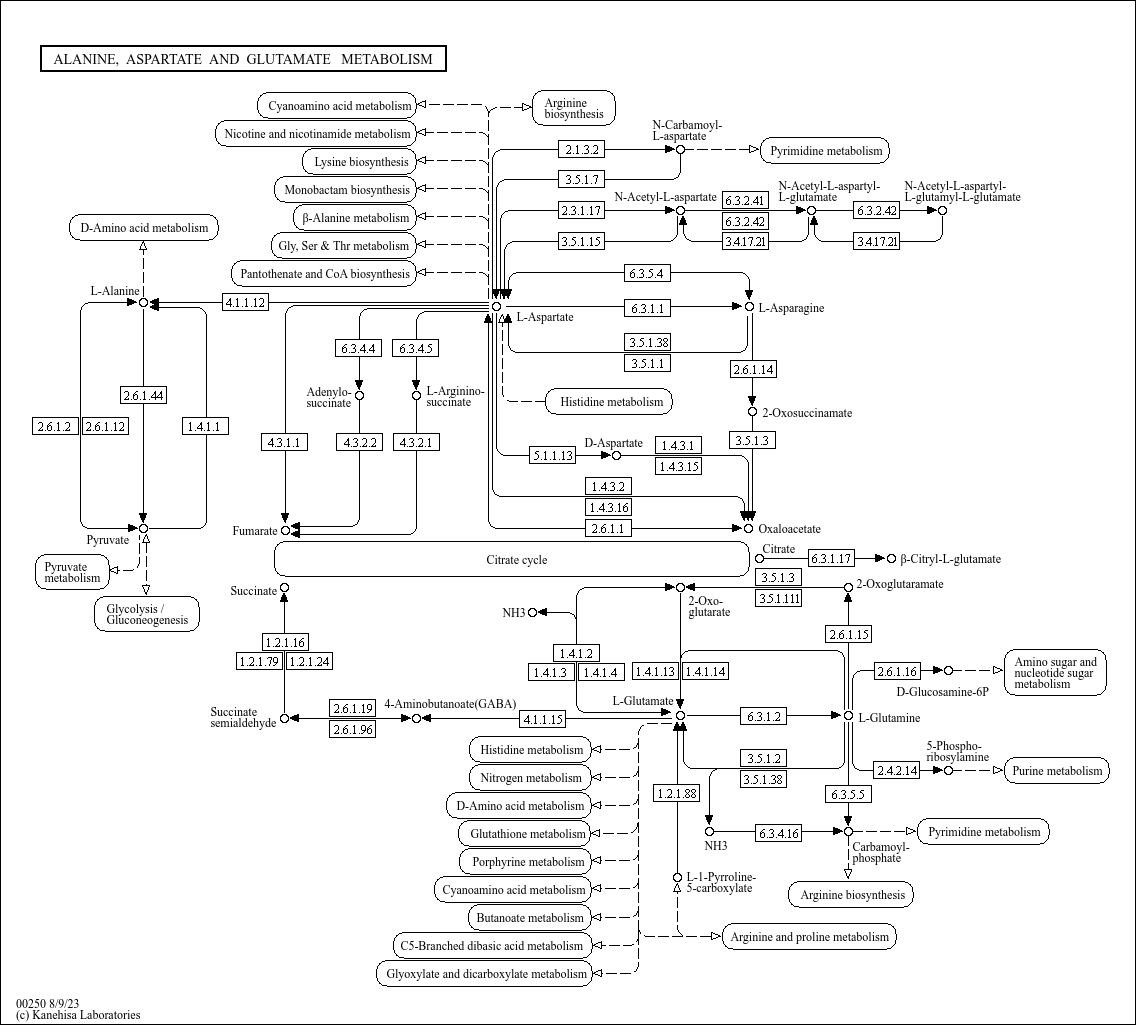


**Figure 1:** Alanine aspartate and glutamate pathway


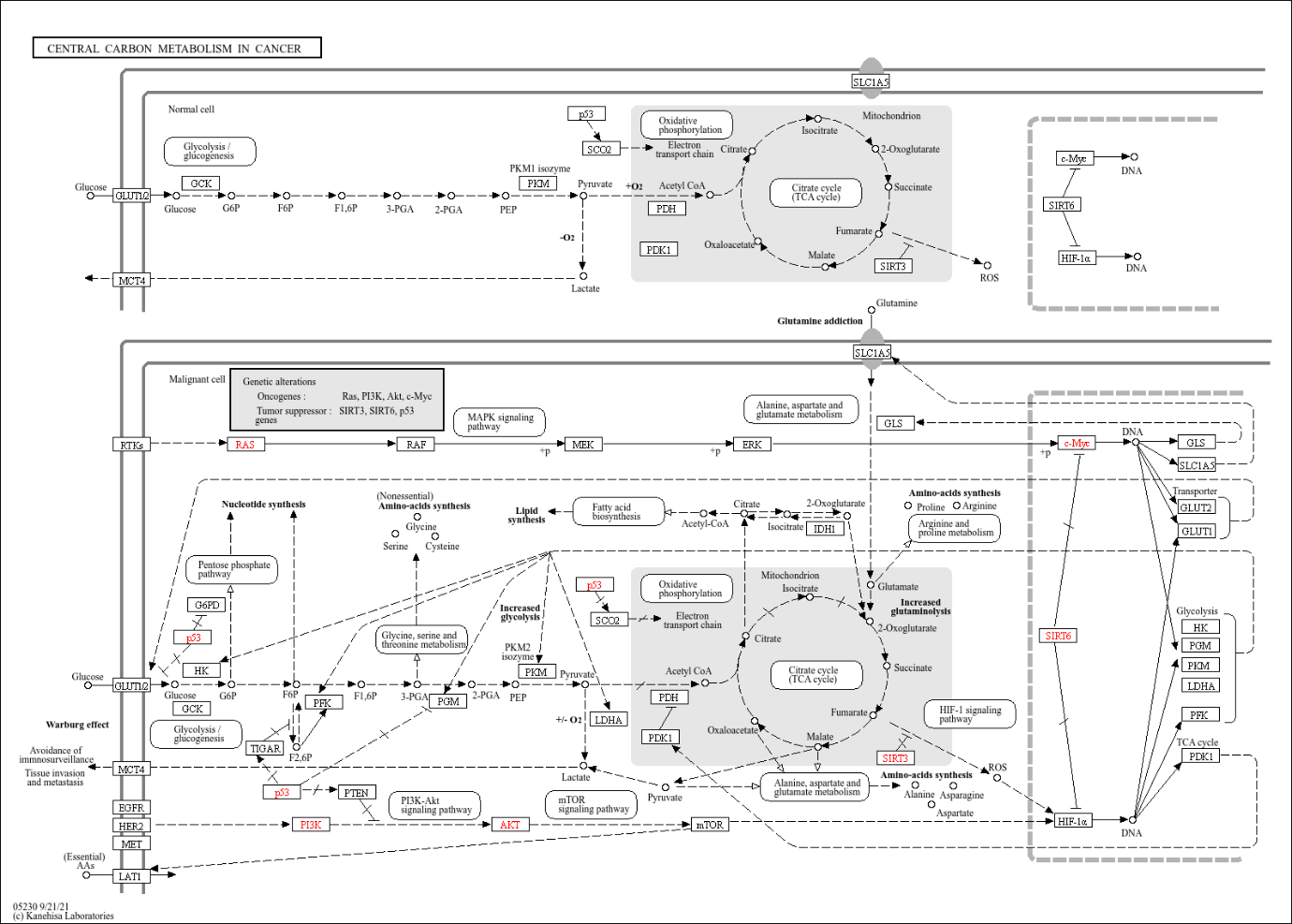


**Figure 2:** Central carbon pathway

**Figure 3:** Gas chromatography-mass spectrometry of the methanolic extract of *G. max* after the application of Photon. Retention time (RT): 0.00 – 23.20
